# Colloidal Stability, Sedimentation, and Aggregation of Crystalline Two-Dimensional Crumpled Birnessite Flakes, Their Dye Adsorption and Immune Cell Response

**DOI:** 10.1021/acs.langmuir.4c03802

**Published:** 2025-02-14

**Authors:** Mary Qin Hassig, Adam D. Walter, Vanessa R. Morris, Yucheng Zhu, Ahmed M. H. Ibrahim, Abijah Gordon, Mohamed A. Ibrahim, Hao Cheng, Hussein O. Badr, Michel W. Barsoum

**Affiliations:** † Department of Material Science and Engineering, Drexel University, Philadelphia, Pennsylvania 19104, United States; ‡ Department of Chemistry, Physics and Materials Science, Fayetteville Statue University, Fayetteville, North Carolina 28301, United States; § Department of Chemical Engineering, Stanford University, Stanford, California 94305, United States

## Abstract

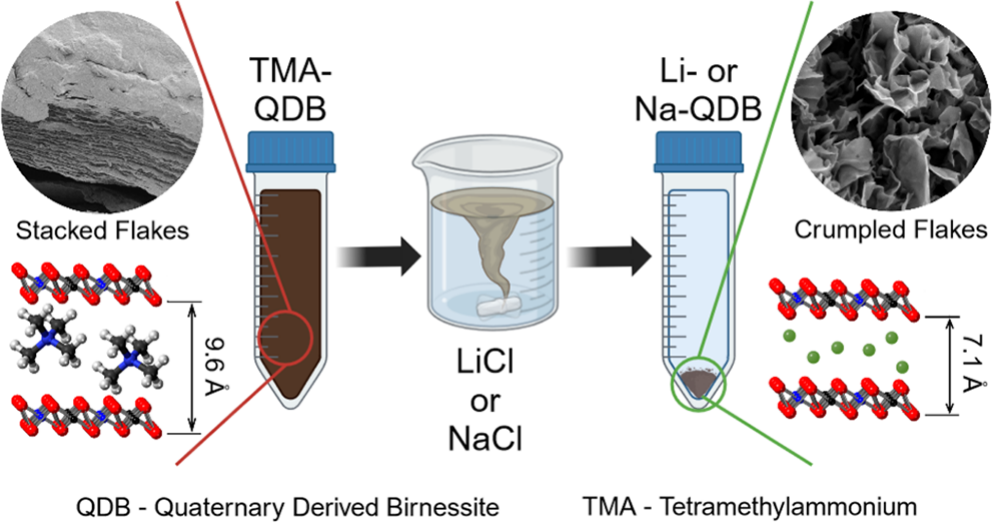

Highly crystalline two-dimensional (2D) flakes of birnessite, a polymorph of manganese oxide with a MnO_2_ chemistry, were synthesized by reacting manganese oxide, Mn_3_O_4_, at 80 °C with aqueous solutions of tetramethylammonium hydroxide (TMAH) for tens of hours. Their colloidal stability, aggregation, and sedimentation were studied as a function of ionic strengths of Na^+^ and Li^+^ cations. After reaction, a water-based stable colloidal suspension (ζ-potential ∼ −31 ± 1 mV) was obtained. Mixing the colloidal suspension with a LiCl or NaCl aqueous solution resulted in the sedimentation of crumpled flakes, as evidenced by electron microscopy (transmission and scanning). Concomitant with the sedimentation, the TMA^+^ cations present after synthesis are exchanged by the alkali ions, as evidenced by a decrease in the *d*-spacings between the 2D sheets illustrated by X-ray diffraction (XRD). Both Na and Li uptakes were quantified by elemental analysis via inductively coupled plasma tandem mass spectrometry, giving Li_0.17_Mn_0.96_O_2_ and Na_0.16_Mn_0.96_O_2_. Rhodamine 6G dye was also studied as a sedimentation agent, resulting in a maximum uptake of 550 mg (1.15 mmol) of dye per g of birnessite. To explore the immune response of the Li^+^-intercalated crumpled flakes, the activation of antigen-presenting cells by the flakes was investigated. It was found that the immune cells were slightly activated in a dose-dependent manner, indicating that the materials may have good biocompatibility and thus possibe applications in healthcare.

## Introduction

Manganese oxide, MnO_*x*_, comes in several polymorphs, including α (hollandite), β (pyrolusite), γ (intergrowth), and λ (defect spinel) and has found many uses in a wide range of fields.^1^ MnO_*x*_ compounds have found applications in energy storage—as electrodes in batteries and supercapacitors,^2−6^ oxidation catalysis,^2,7,8^ solar cells,^9^ heavy metal adsorption,^10,11^ and biosensing/drug delivery.^12^ Of special interest to this work is birnessite, also known as δ-MnO_2_^13,14^ or manganous manganite.^15,16^ The name originates from the area where a large natural deposit of birnessite was discovered—Birness, Scotland. When found in nature, birnessite is layered but poorly crystalline.^7,17^

Typically, layered MnO_2_ is synthesized by reducing potassium permanganate (KMnO_4_),^8,13,18^ oxidizing Mn-hydroxides with O_2_ or Cl_2_ in hydroxide solutions,^13,19^ topotactic oxidation of Mn salts in combination with an alkaline solution,^20−22^ or by hydrothermally treating Mn compounds, such as salts or oxides.^19,23,24^ In 2008, Kai et al. were one of the first groups to synthesize monolayer or few-layered birnessite through coprecipitation.^1^ Their method entailed reacting hydrogen peroxide with the quaternary ammonium, quat, hydroxides, of tetramethylammonium hydroxide (TMAH), or tetrabutylammonium hydroxide (TBAH) and manganese(II) chloride in aqueous solutions to obtain single-layer, colloidal, MnO_2_ nanoflakes.^1^ Another method used to synthesize monolayer δ-MnO_2_ is to exfoliate layered, protonic, Mn-oxide precursors in combination with TBAH. As the Mn precursor is etched, the large TBA^+^ cation is intercalated between the layers, and in turn aids the multilayer separation.^25,26^

The above-mentioned delamination technique is common among layered materials, including clays, and other metal oxides, due to the presence of preexisting interlayer cations, which are readily ion exchanged.^25,27,28^ These counter cations help maintain the charge neutrality as the layers are generally negatively charged.^27−29^ Similar to the structure of other layered metal oxides, birnessite layers are made from edge-sharing MnO_6_ octahedra with cations occupying the interlayer space.^25,30,31^ Natural birnessite samples have been found with many different cations in interlayer space including Na^+^, Li^+^, K^+^, Ca^2+^, Mg^2+^, and Ba^2+^.^31^ This large variety of interlayer cations, combined with the ease of cation exchange, allows birnessite to lend itself to applications such as water treatment^32−35^ and energy storage.^36−38^

In previous work, we showed that by simply reacting several water-insoluble Mn compounds, including Mn_3_O_4_, Mn_2_O_3_, MnB, Mn_5_SiB_2_, and Mn_2_AlB_2_, with aqueous quat solutions, at near ambient conditions, resulted in large, crystalline, 2D, birnessite-based single flakes.^39,40^ These flakes are further referred to as quaternary derived birnessite (QDB). This bottom-up method is simple, scalable, less expensive, and less time-consuming than top-down methods and ultimately results in a more crystalline product.^39,40^ For this work, Mn_3_O_4_ was chosen as the precursor because it is relatively inexpensive, earth-abundant, and nontoxic.^41−43^ Additionally, our processing conditions were chosen, including the precursor and solvent, as they lead to the highest conversion rates.

QDBs are part of a larger family of materials we discovered recently that we are referring to as hydroxide-derived nanomaterials. This family consists of nanomaterials made with several transition metal precursors and hydroxide solution combinations.^39,40,44−47^ A main member of this family is quantum-confined, one-dimensional lepidocrocite (1DL) made by reacting binary and ternary Ti compounds with TMAH.^46−48^ 1DLs have shown the ability to uptake various cations,^48−51^ modifying their structure and some of their properties as a result of this exchange.

This work’s purpose was threefold. The first was to shed light on what happens when QDB colloidal suspensions with TMA^+^ cations are immersed in LiCl or NaCl aqueous solutions, also referred to as Li-QDB and Na-QDB, respectively. We find that the salt solutions result in a rapid deflocculation of the 2D sheets and their crumpling. The second was to explore their potential in water remediation by adsorbing the dye rhodamine 6G (Rh6G). The third is to evaluate the biocompatibility of the QDBs by studying the activation of antigen-presenting cells by the flakes as TMAH is known for being a hazardous chemical with fatal consequences if handled improperly or ingested.^52,53^

## Results and Discussion

**Figure 1:**
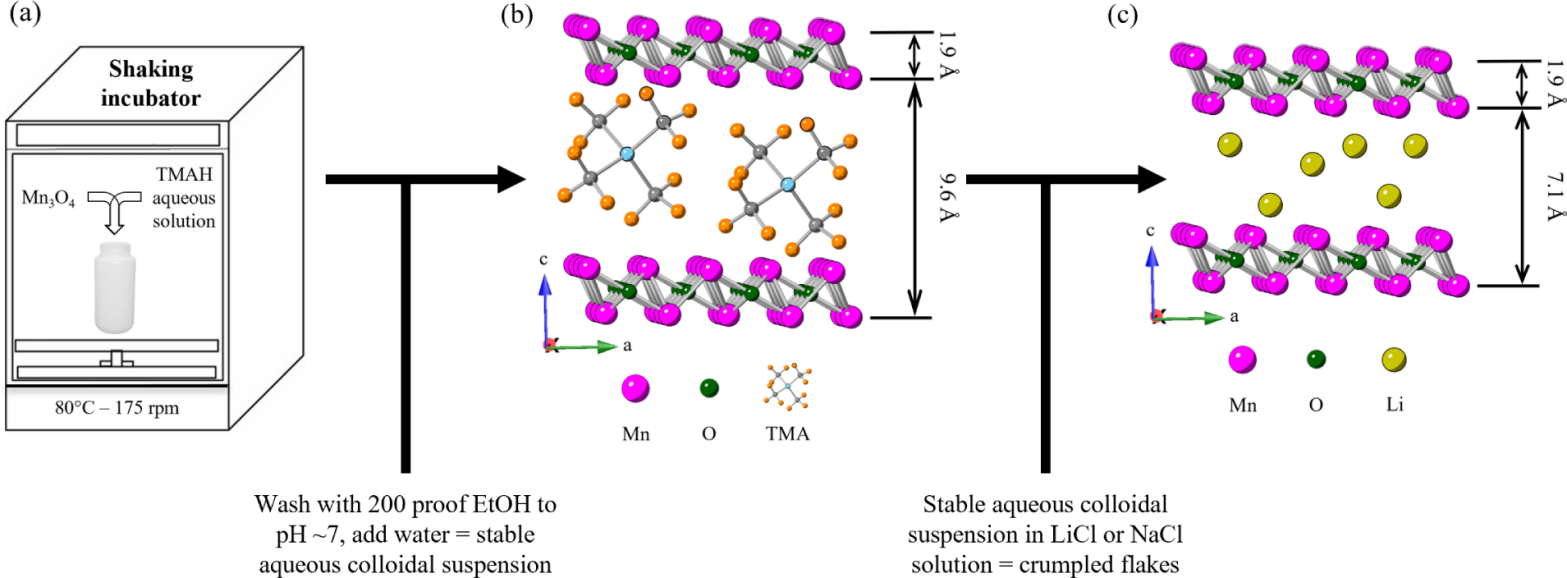
Scalable synthesis of 2D birnessite flakes. Schematic of (a) temperature-controlled shaking incubator used to convert Mn_3_O_4_ precursor powder into 2D birnessite flakes. (b) Isometric side view of relaxed DFT structure of birnessite.^40^ (c) Isometric side view of ion exchanged birnessite. The schematics are roughly to scale.

The details for synthesizing the 2D birnessite flakes can be found at the end of this paper, in the “Experimental Procedures” section. In short, we combined 1 g of precursor Mn_3_O_4_ powder with 10 mL of TMAH, 25 wt % aqueous solution, and heated the mixture to 80 °C in a shaking incubator oven (Figure 1a) for 4 days, d. Post-reaction and cooling, the samples were washed several times with ethanol (EtOH) until the excess base was removed (pH ≈ 7). Adding deionized (DI) water at this juncture results in a stable aqueous colloidal suspension of 2D birnessite flakes with TMA^+^ cations intercalated between the layers (Figure 1b). Some of these suspensions were then mixed with various ionic solutions overnight, washed with DI water, and vacuum filtered. The new ion-intercalated structure can be seen schematically in Figure 1c. The details of the material characterization techniques of filtered colloid 2D flakes can be found in our previous publications.^39,40^

Immediately after the colloidal suspension was mixed with NaCl or LiCl solutions, the 2D flakes fell out of the suspension and settled to the bottom of the reaction bottle. Comparable to the interactions between other layered clays,^54^ MXenes^55^ and 1DLs^48^ with salt ionic solutions, the cations electrostatically adsorb onto the birnessite sheets that are negatively charged. Following the Derjaguin–Landau–Verwey–Overbeek (DLVO) theory, which is traditionally used with spherical particles, not flakes, the alkali ions are presumed to form an electric double layer on the surface of the flakes, which varies in thickness based on the solution’s ionic strength. The higher the ionic strength, the thinner the double layer, which decreases the Debye length, which, in turn, decreases the electrostatic repulsion between the faces of the flakes. This reduction in repulsion allows for the van der Waals attraction forces to now outweigh the repulsive forces and lead to aggregation.^55−58^ The crumpled flakes are further investigated in the sections below.

### Material Characterization

The samples prepared for structural analyses were mixed with 5 M aqueous salt solutions to result in immediate crumpling. This concentration was chosen as the standard based on our previous ion intercalation work,^46,48^ but it is unnecessarily concentrated, wasting ionic solution and deionized water from additional washing. However, this excess was used to ensure complete sedimentation and maximum crumpling.

**Figure 2:**
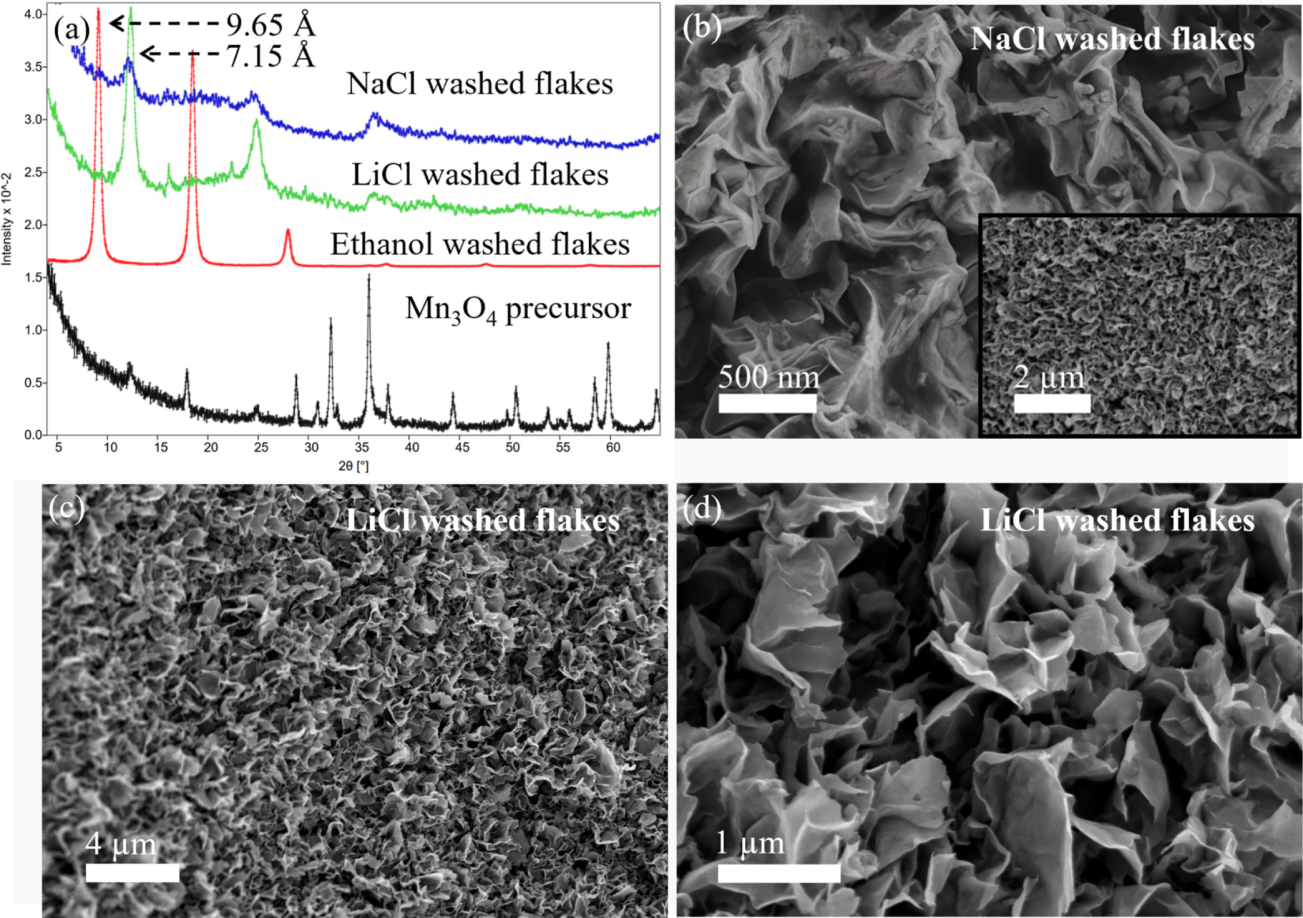
(a) XRD patterns of Mn_3_O_4_ precursor powder (bottom black curve), as well as samples reacted in TMAH aqueous solutions at 80 °C for 4 d and then washed with solvent/solution labeled in panel. Horizontal arrows denote *d*-spacing values for TMA^+^ and Li^+^ ion-intercalated flakes. (b)–(d) SEM micrographs—at various magnifications—of crumpled flakes deflocculated with, (b) NaCl or (c)–(d) LiCl aqueous solutions. Inset in (b) is the same as (b) but at a lower magnification.

Figure 2 shows the X-ray diffraction patterns of the precursor, EtOH-washed flakes, and those washed with LiCl or NaCl, from bottom to top, respectively, as labeled in Figure 2a. After washing with EtOH (red pattern in Figure 2a), six 00L peak reflections are present, indicating high order along the c direction. In all cases, precursor peaks in the patterns of the reacted material are absent, and only 00L peaks of the birnessite flakes are present.

At ≈9.65 Å, the *d*-spacing of the EtOH-washed samples was slightly higher than our previous value of 9.4 Å but in line with other published work on TMA^+^ intercalated birnessite.^1,30,59^ Once the colloidal suspension is mixed with the other ionic solutions, the basal spacing decreases to ≈7.15 Å. This shrinkage confirms the success of the ion exchange, as Li^+^ and Na^+^ are both smaller in diameter (1.52 and 2.04 Å, respectively)^56^ than the 5.6 Å value of the TMA^+^ cation.^60^ Interestingly, if we assume, as we did earlier, that the thicknesses of the MnO_2_ layers are ≈1.9 Å, then the hydrated diameters of the Li^+^ and Na^+^ cations would be ≈5.25 Å. This value falls between the established values of 4.26 and 4.82 Å for average Li^+^ and Na^+^ hydrated diameters^61^ and absolute hydrated diameters of 5.30 Å^62^ and 6.24 Å.^63^ It follows that in this case, the cations are most probably partially hydrated.

Figure 2b–d shows scanning electron microscope (SEM) micrographs of crumpled flakes with NaCl or LiCl aqueous solutions. Comparing these SEM micrographs to the ones obtained after EtOH-only washing (Figure S1)^39,40^ evidences the crumpling quite clearly.

**Figure 3:**
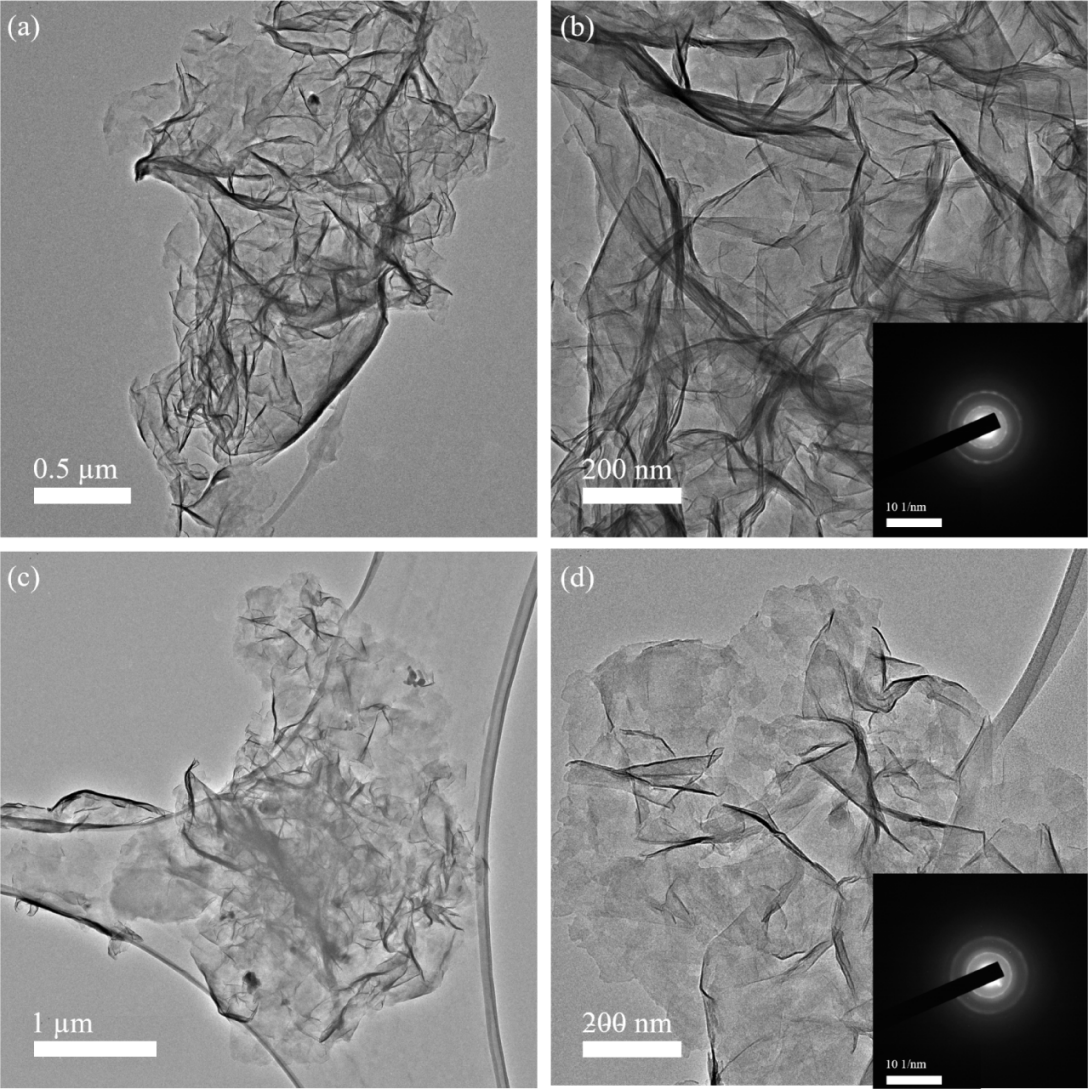
(a) and (c) TEM micrographs of Mn_3_O_4_ powder after reaction with TMAH aqueous solutions at 80 °C for 4 days, washed with EtOH, and then treated in LiCl aqueous solution (b) and (d) High magnification images of flakes captured in (a) and (c), respectively. Insets in (b) and (d) show corresponding SAED patterns.

When transmission electron microscope (TEM) micrographs of LiCl-washed flakes (Figure 3) are compared with those only washed with EtOH, shown in our previous work,^40^ it is apparent that the former are much more crumpled as evidenced by the multiple folds and creases visible here. The selected area electron diffraction (SAED) patterns, shown as insets in Figure 3b,d, have two prominent rings with *d*-spacings of ≈2.5 Å and ≈1.5 Å that can be ascribed to the d_100_ and d_110_ spacings of hexagonal birnessite, respectively. These values are consistent with our previous findings^39,40^ and show that the flakes maintain their structure after crumpling.

### Colloidal Stability

As produced, QDB colloids in water are stable for at least 12 months without settling with a zeta potential of −31 ± 1 mV.^39^ However, when a LiCl solution with a concentration of >20 mM is added to the colloidal suspensions (Figure S2), flocculation and sedimentation are observed and are reinforced by the lack of Tyndall effect in Figure S3. The pictures shown in Figures S2 and S3 were taken immediately after the LiCl solutions were added, which lead us to conclude that the deflocculation is instantaneous and requires no stirring. The same is true when 5 M are added.

### Quantitative Analysis of Cation Exchange

In the remainder of this paper, we present results that, in principle, could lead to applications. This is especially true here, given the low cost of our precursor and the ease and simplicity of scaling up our process. To understand the degree of cation exchange, the as-synthesized QDB, Li-QDB, and Na-QDB were dissolved in 12 M HCl and analyzed via an ICP-MS triple quadrupole (ICP-QQQ). The Li/Mn and Na/Mn atomic ratios were determined to be 0.18 ± 0.04 Li/Mn and 0.17 ± 0.01, respectively, after removing the Li baseline. Neither sample contained detectable amounts of any other cation. Since birnessite is cation deficient,^1,4,19^ we determine the overall formulas to be Li_0.17_Mn_0.96_O_2_ and Na_0.16_Mn_0.96_O_2_. The fact that we get essentially the same formula for both cations is gratifying since it implies that the net negative charges on the MnO_2_ sheets are not a function of the cation. Presumably, the negative charge is determined during the reaction with TMAH.

**Figure 4:**
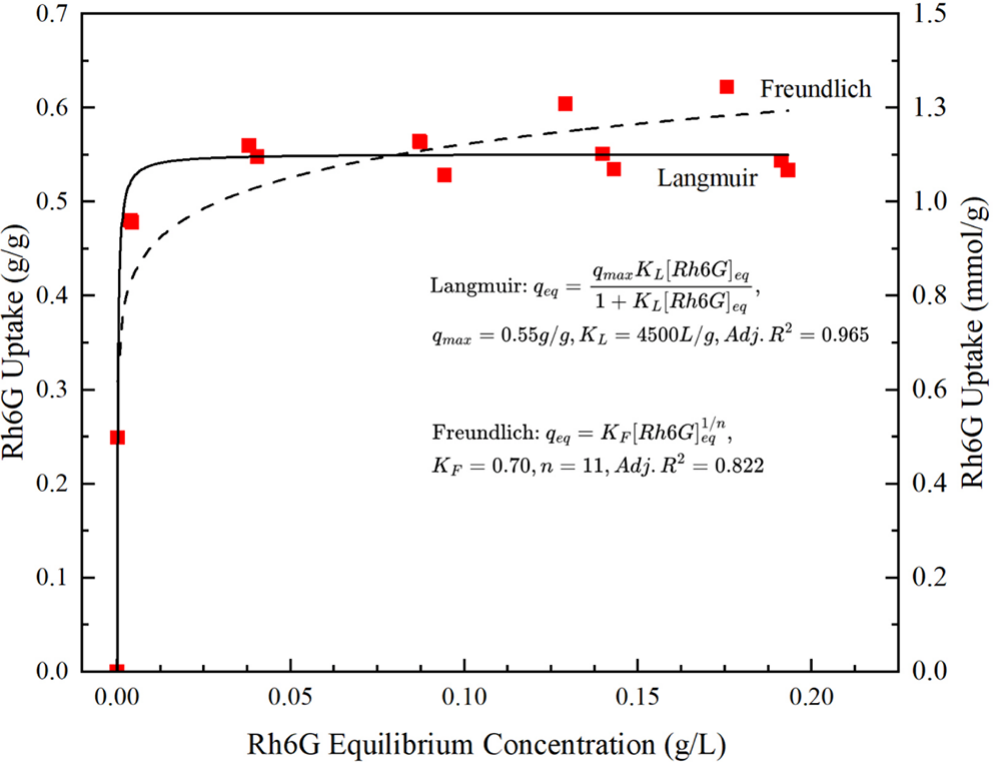
Room temperature adsorption of Rhodamine 6G with fits to the Langmuir (solid line) and Freundlich (dashed line) isotherm relation. The corresponding fit parametes are shown in panel's center. Left hand y-axis denotes values as g/g; right-hand axis the same reuslts, but as mmol/g of QBD.

Utilizing the ion exchange capacity of QDB is best visualized in the adsorption of toxic waste dyes. Manganese oxide is widely used as an adsorbent material.^64^ In some cases, it can also act as an oxidation catalyst under acidic conditions.^65^ While a promising avenue, the latter is outside the scope of this work. This section deals with adsorption of the toxic waste dye rhodamine 6G, Rh6G, at room temperature, RT, on unmodified QDB colloidal at a pH of ∼10.5. As seen in Figure 4, the adsorption isotherm can best be described as Langmuir-type, with a maximum uptake (*q*_max_) of 0.55 g/g and a Langmuir constant (*K*_L_) of 4,500 L/g. A Freundlich-type isotherm was also explored resulting in a Freundlich constant or distribution factor (*K*_F_) of 0.70 g/g and a correction factor (*n*) of 11.^11^ This fit was less successful, and based on the comparison of the assumptions of each model, the Langmuir-type (with monolayer coverage) was determined to be the best model for this system. The equilibrium value obtained, 550 mg/g, is significant because it outperforms both activated carbon (28 mg/g)^66^ and cation-exchangeable clay montmorillonite (376 mg/g).^66^ It is also worth noting that less crystalline birnessite only adsorbs Rh6G to a maximum of 94 mg/g,^67^ while another study showed adsorption of rhodamine B, a similar dye to Rh6G, to a maximum of 45 mg/g.^65^

According to the ICP-QQQ analysis, the as-synthesized QDB is 42 wt % Mn. Knowing this, combined with the fact that the amount of Rh6G in the exchanged QDB is 1.15 mmol/g of birnessite (Figure 4, right axis) gives a Rh6G to Mn atomic ratio of 0.15, resulting in a formula of (Rh6G)_0.14_Mn_0.96_O_2_. According to the Li-QDB and Na-QDB formulas above, these results track. It is apparent that the cation exchange capacity increases slightly for the smaller cations; however, it the increase is not substantial. Averaging the stoichiometric ratios of the other cations, it is possible to assume that the initial formula of QDB is TMA_0.16_Mn_0.96_O_2_ which results in ≈13 wt % TMA^+^. Coincidentally or not, this value is *quite close* to the TMA^+^ content in 1DL.^68^

### Activation Status of Antigen-Presenting Cells

The crumpled flakes may also have biomedical applications, which often require materials to be biocompatible. At a low concentration of only 16.67 mg/kg, TMAH is fatal in both contact with the skin and if swallowed. TMAH is also known to cause severe eye damage, damage to the central nervous system after a single exposure, and damage to the liver and thymus with extended or long-term exposure.^52,53^ Swapping the TMA^+^ cation with known biocompatible cations such as Li^69^ allows for the study of the biocompatibility of the QDB itself. The absence of TMA^+^ in the Li-QDB samples was established through their compatibility with the living cells in the work described below and by the absence of a nitrogen, N 1s, signal in the survey spectra from X-ray photoelectron spectroscopy, XPS, shown in Figure S4.

Immune cell response to biomaterials is a good indicator of their biocompatibility *in vivo*. Antigen-presenting cells, APCs, play important roles in immune responses. Bone marrow-derived dendritic cells (BMDCs), representative APCs, were selected for the study, and the cells were treated with 3 concentrations of the Li-QDB flakes, viz. 10 μg/mL, 50 μg/L, and 100 μg/mL. Lipopolysaccharide, LPS, a natural adjuvant was used as a positive control for BMDC activation. Interestingly, the colloid concentration does not appear to play a strong role. Not surprisingly, LPSs had the greatest effect.

**Figure 5:**
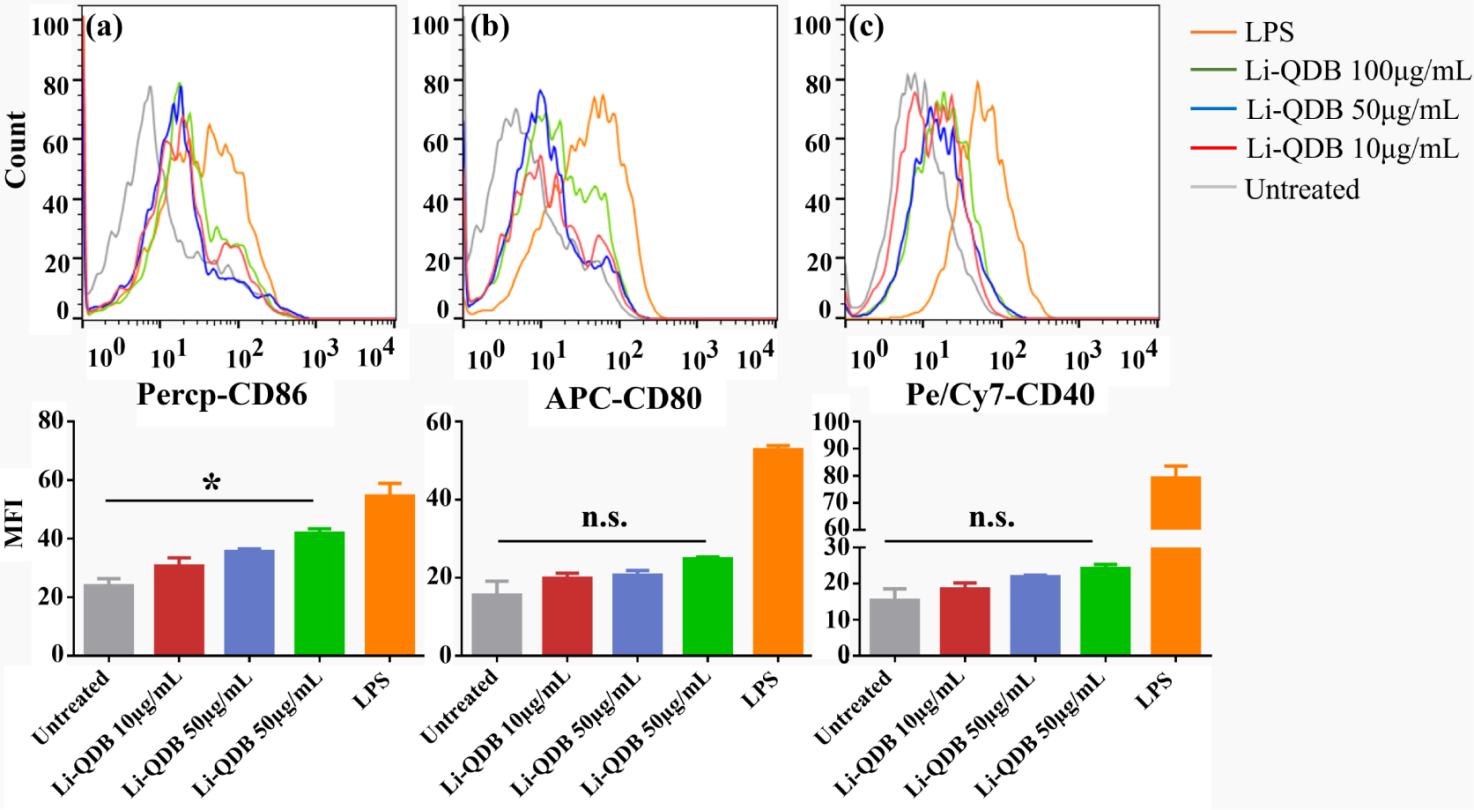
Effect of crumpled flakes on the activation of BMDCs. Cells were treated under different concentrations of the flakes for 24 h before analysis of their expression of costimulatory molecules. Untreated cells and cells treated with LPS were used as negative and positive controls. Flow cytometry histograms and MFI of CD86 (a), CD80 (b), and CD40 (c).

After 24 h, the cells were collected and analyzed using flow cytometry for their expression of costimulatory molecules, CD86, CD80, and CD40 (Figure 5). The histogram of flow cytometry and mean fluorescence intensity (MFI) shows that the expression of costimulatory molecules increased slightly with the increase of flake concentrations in a dose-dependent manner. However, compared to the untreated control, the increases are mostly not statistically significant except for the CD86 expression at the high flake concentration of 100 μg/mL (Figure 5).

## Conclusions

Herein we show that deflocculating colloidal suspensions of 2D MnO_2_ birnessite flakes, with 5 M NaCl or LiCl, result in their crumpling as evidenced in XRD patterns, SEM and TEM micrographs. The flakes readily and rapidly adsorbed the dye R6G with a maximum uptake of 550 mg (1.15 mmol) dye per g of birnessite.

Furthermore, after investigating the effect of the Li^+^-intercalated flakes on the activation status of antigen-presenting cells, the crumpled flakes were found to be biocompatible as they barely stimulated the expression of costimulatory molecules, supporting further studies of these flakes as biomaterials.

## Experimental Procedures

### Material Synthesis and Processing

Synthesis of birnessite 2D flakes was carried out following our recently developed protocol, in which we heat Mn_3_O_4_ powders in TMAH aqueous solutions for a few days under ambient pressure.^39,40^ In a typical experiment, 1 g of Mn_3_O_4_ powder (97%, Strem Chemicals Inc., Newburyport, MA, USA) and 10 mL TMAH aqueous solution (25 wt % in water, 99.9999%, Alfa Aesar, Haverhill, MA, USA) were placed in a polypropylene bottle and shaken in a shaking incubator (211DS 49L, Labnet International, Inc., Edison, NJ, USA) at 80 °C for 4 d (Figure 1a). After reaction, the resulting slurry was rinsed with EtOH (200 proof, DECON Lab, Inc., King of Prussia, PA, USA) for a total of 5 washing cycles to remove any unreacted TMAH base. In each cycle, 40 mL EtOH was added to the sediment, the mixture was shaken briefly, centrifuged at 3500 rpm for 2 min, and the supernatant discarded. Finally, when the pH dropped down to ∼7, 30 mL of DI water was added to the sediment, the mixture was vortex shaken for 5 min, and centrifuged at 3500 rpm for 30 min. Typically, this procedure resulted in a stable colloidal suspension of ∼10 mg/mL. Filtering the latter produced free-standing filtered films (Figure S5) that were air-dried overnight before further characterization.

To prepare the 2D flakes intercalated with the cations, 50 mL of 5 M LiCl or 5 M NaCl aqueous solution was added to 20 mL of the colloidal suspension obtained above. The mixture was stirred at RT for 24 h. This resulted in flocculation. The mixture was washed for 3 cycles with DI water to remove any unreacted salts. After filtration, the Li^+^- or Na^+^-intercalated powders were obtained and left to dry in open air overnight before further characterization.

### X-ray Diffraction

X-ray diffraction (XRD) patterns on air-dried filtered films/powders were acquired by using a diffractometer (Rigaku SmartLab, Tokyo, Japan) operated with Cu Kα radiation. The samples were scanned in the 2–65° 2θ range by using a 0.02° step size and a dwell time of 1 s per step.

### Scanning Electron Microscopy

Micrographs were obtained using a SEM (Zeiss Supra 50 VP, Carl Zeiss SMT AG, Oberkochen, Germany) operated using an in-lens detector, 30 μm aperture, and an accelerating voltage of 3 kV.

### Transmission Electron Microscopy

A TEM (JEOL JEM2100F field-emission TEM Peabody, MA, USA) was used to image the flakes and obtain selected area electron diffraction (SAED) patterns. The TEM was operated at 200 keV and has an image resolution of 0.2 nm. Images and diffraction patterns were collected on a charge-coupled device camera, a CCD (Gatan USC1000). TEM samples were prepared by first diluting the colloidal suspensions before drop casting a few drops of the latter on a carbon-coated, lacy carbon copper TEM grid.

### Inductively Coupled Plasma Tandem Mass Spectrometry (ICP-QQQ)

Powder samples were dissolved at 80 °C in 12 M HCl (trace metals grade, Thermo Fisher Scientific, Waltham, MA, USA) to a concentration of 0.5 g/L (500 ppm). An aliquot of 1 mL was taken and diluted to 50 mL with ultrapure water (i.e., a 50× dilution to 1 ppm). The sample was introduced into an ICP-QQQ (8900 with SPS 4 autosampler, Agilent, Santa Clara, CA, USA). The samples were run in helium, He, mode using ultrapure water as the blank, utilizing a mixed Mn/Li/Na ICP-MS calibration standard (ICM-103, Agilent Technologies, Santa Clara, USA) to determine their concentration. A few runs were carried out initially to determine the background level of Li and Na. We measured the atomic ratios of Li and Na to Mn in the QDB and found them to be 0.044 and 0, respectively. We attribute Li to trace contamination from the instrument. Henceforth, this value will be used as the Li baseline to subtract from all samples. Li is a “sticky” element, in that once analyzed it will remain in the instrument in non-negligible concentrations. We also hereby acknowledge the difficulty in analyzing trace levels of Na due to high levels of environmental Na; however, in this work, we completed all analysis at >100 of ppb of Na.

### Dye Adsorption

A stock aqueous solution of Rh6G (as received, 99%, Thermo Fisher Scientific, Waltham, MA, USA) was prepared to a concentration of 0.5 g/L. Six vials of 2 mL of 1 g/L QDB colloidal suspensions were prepared. To each, 1 to 6 mL of 0.5 g/L dye solution was added (see Table 1). The mixtures were allowed to shake at 200 rpm in glass vials for 1 h to ensure that kinetic equilibrium was reached. After 1 h, the mixture was filtered through a 0.45-μm PTFE syringe filter. The resulting solutions were analyzed in poly(methyl methacrylate) cuvettes with a 1 cm path length using an ultraviolet–visible spectrophotometer (Cary 60, Agilent Technologies, Santa Clara, CA, USA) with a scan rate of 300 nm/min. Samples were tested in triplicate.

**Table 1:** Sample Information for Rh6G RT Adsorption Isotherm Shown in Figure 4

0.5 g/L Dye (mL)	1 g/L Colloid (mL)	DI Water (mL)
1	2	7
2	2	6
3	2	5
4	2	4
5	2	3
6	2	2

### X-ray Photoelectron Spectroscopy (XPS)

A spectrometer (VersaProbe 5000, Physical Electronics, Chanhassen, MN, USA) was used to obtain the XPS spectra. The samples were mounted on an Al stub via carbon tape and analyzed without Ar^+^ sputtering. Monochromatic Al K_α_ X-rays, with a pass energy of 27.0 eV, a step size of 0.50 eV, a 0.1 s step time, and a spot size of 200 μm, were used to irradiate the sample surfaces. Five scans were obtained for each region. The CasaXPS version 2.3.23PR1.0 software was used for analysis. The obtained spectra were calibrated by setting the C–C binding energy peak to 285.0 eV.

### Activation of Antigen-Presenting Cells

BMDCs were cultured based on an established protocol.^70^ Briefly, the tibias and femurs from female C57BL/6J mice were isolated and placed in 70% EtOH for 2–3 min to sterilize them. The ends of the bones were then cut, and the marrow was flushed with RPMI 1640 medium using a 29G syringe in a clean Petri dish. The cells were dissociated by resuspending with a pipet tip. The cells were then centrifuged, and red blood cells (RBCs) were removed using an RBC lysis buffer (BioLegend, San Diego, CA, USA). The resulting cells were cultured in 60 mm Petri dishes in RPMI 1640 with 10% FBS (ATCC, Manassas, VA, USA), 1% penicillin/streptomycin (ATCC, Manassas, VA, USA), 50 μM 2-mercaptoethanol (Thermo Fisher, Waltham, MA, USA), and 20 ng/mL of GM-CSF (PeproTech, Rocky Hill, NJ, USA) at a concentration of 2 × 10^6^ cells per 5 mL medium. On day 3 of the culture, 2 mL of medium per dish was removed and replaced with 3 mL of fresh medium. On day 6, the loosely attached cells were replated with 5 × 10^5^ cells in 1 mL of medium in a 24-well low-attachment plate.

For flow cytometry cell analysis, the birnessite 2D sheets were added to the wells on day 7 at concentrations of 10 μg/mL, 50 μg/mL, and 100 μg/mL. Alternatively, 1 μg/mL LPS was added as a positive control. On day 8, the loosely attached cells were detached by gentle pipetting and immunostained to probe CD86, CD80, and CD40 (antibodies were purchased from BioLegend, San Diego, CA, USA) before flow cytometry analysis.

## Supporting Information Available

SEM micrographs and photograph of noncrumpled QDB; photographic comparison of LiCl of varying concentrations with QDB; photograph demonstration of the Tyndall effect with LiCl and QDB; XPS survey spectra (PDF)
