# Supplementary material for: Colloidal Stability, Sedimentation, and Aggregation of Crystalline Two-Dimensional Crumpled Birnessite Flakes, Their Dye Adsorption and Immune Cell Response
Source: Langmuir. 2025 Feb 14;41(7):4482–90. doi: 10.1021/acs.langmuir.4c03802 (PMC11866922; doi:10.1021/acs.langmuir.4c03802)
Supplement: Supplementary file 1 — la4c03802_si_001.pdf [file la4c03802_si_001.pdf]

Supporting Information

On the colloidal stability, sedimentation and aggregation of crystalline two-dimensional crumpled birnessite flakes, their dye adsorption and immune cell response

Mary Qin Hassig,[a] Adam D. Walter,[a] Vanessa R. Morris,[b] Yucheng Zhu,[a] Ahmed M. H. Ibrahim,[a] Abijah Gordon,[b] Mohamed A. Ibrahim[a], Hao Cheng,[a] Hussein O. Badr,[a,c*] and Michel W. Barsoum[a*]

[a] Department of Material Science and Engineering, Drexel University, Philadelphia, PA, 19104, USA
[b] Department of Chemistry, Physics and Materials Science, Fayetteville Statue University, Fayetteville, NC, 28301, USA
[c] Department of Chemical Engineering, Stanford University, Stanford, California, 94305, USA
*Corresponding Authors: Michel W. Barsoum (barsoumw@drexel.edu), Hussein O. Badr (hbadr@stanford.edu)

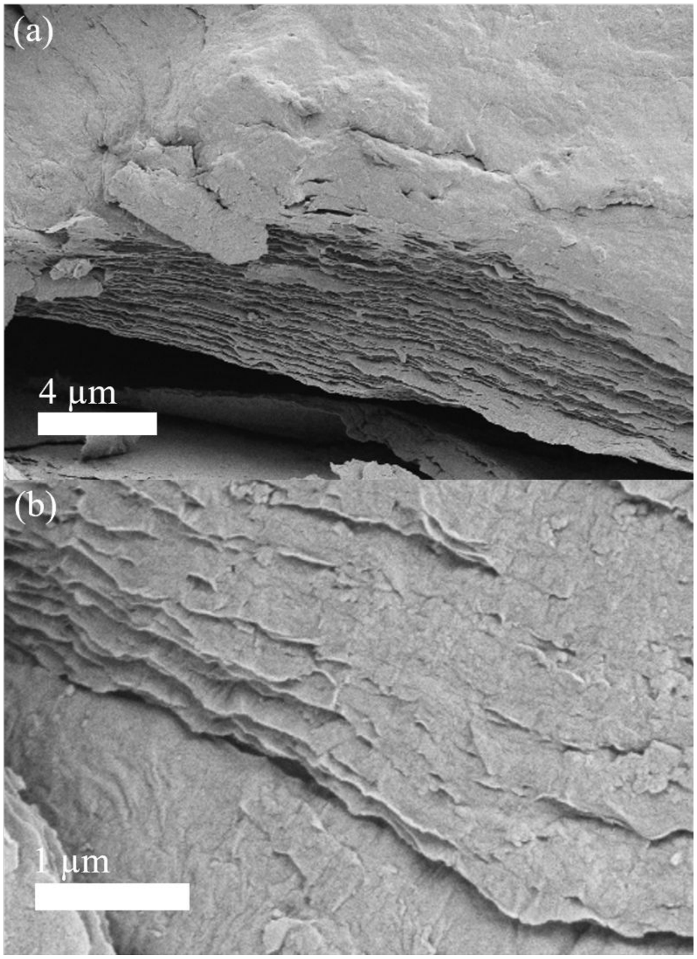

Figure S1: (a)-(b) SEM micrographs – at various magnifications – of non-crumpled QDB flakes.

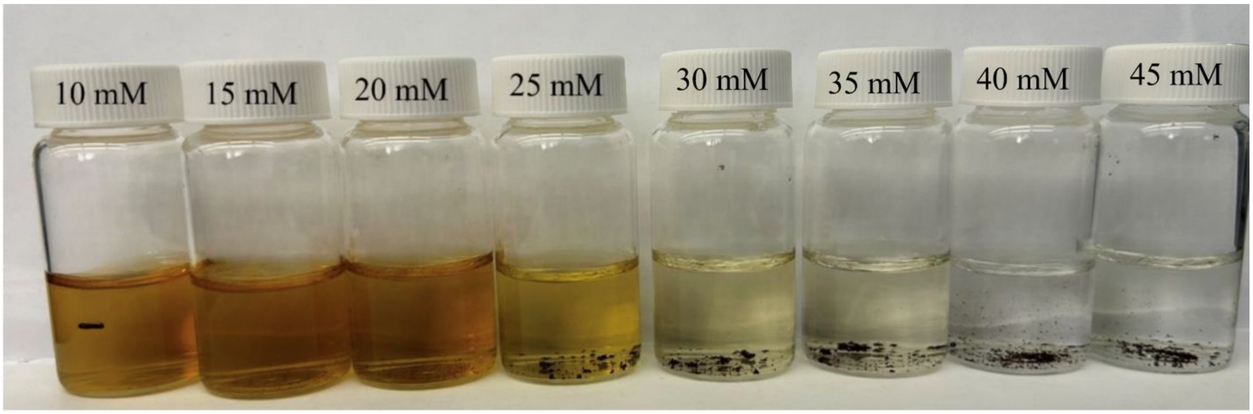

Figure S2: QDB colloidal suspensions with varying LiCl concentrations showing flocculation of colloidal suspension at concentrations > 20 mM. LiCl concentrations, from left to right, are 10 mM and increase in 5 mM increments up to 45 mM.

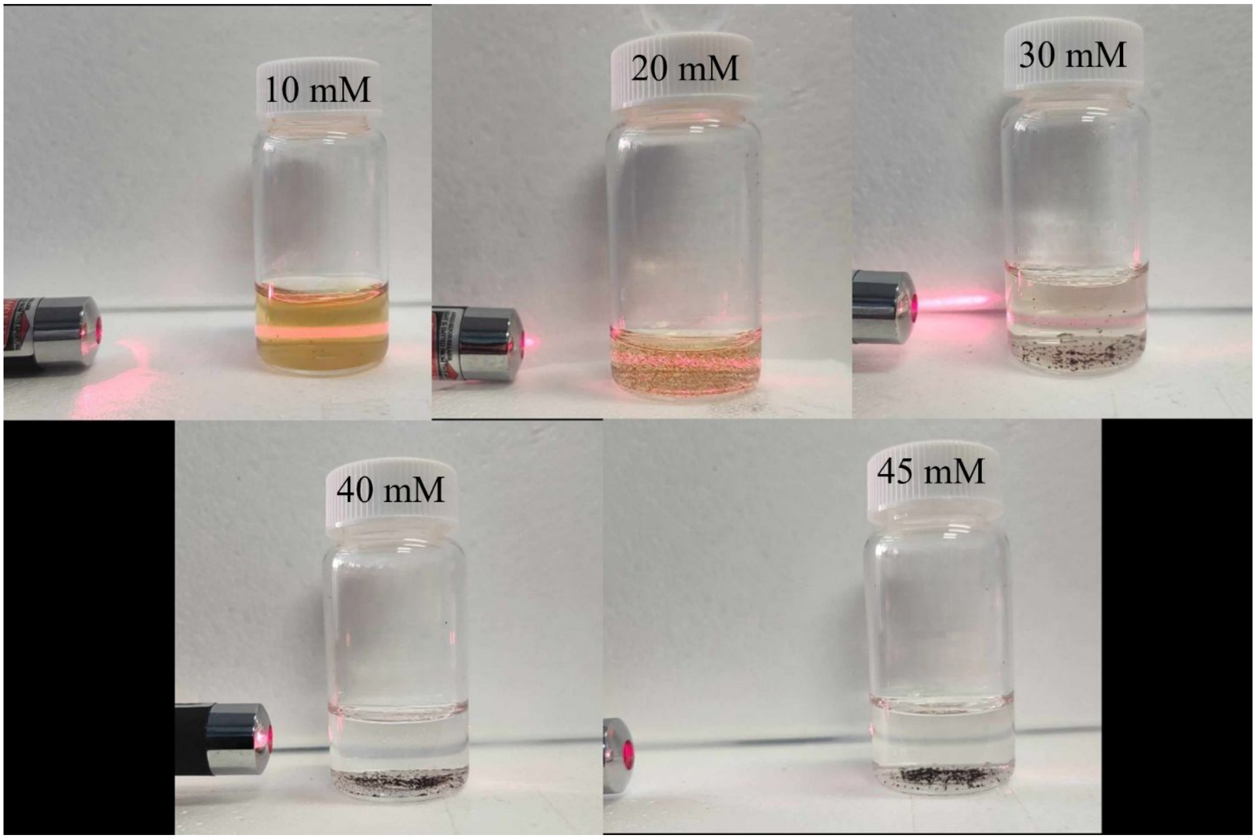

Figure S3: QDB colloidal suspensions with varying LiCl concentrations showing flocculation of colloidal suspension at concentrations > 20 mM displaying the lack of Tyndall effect.

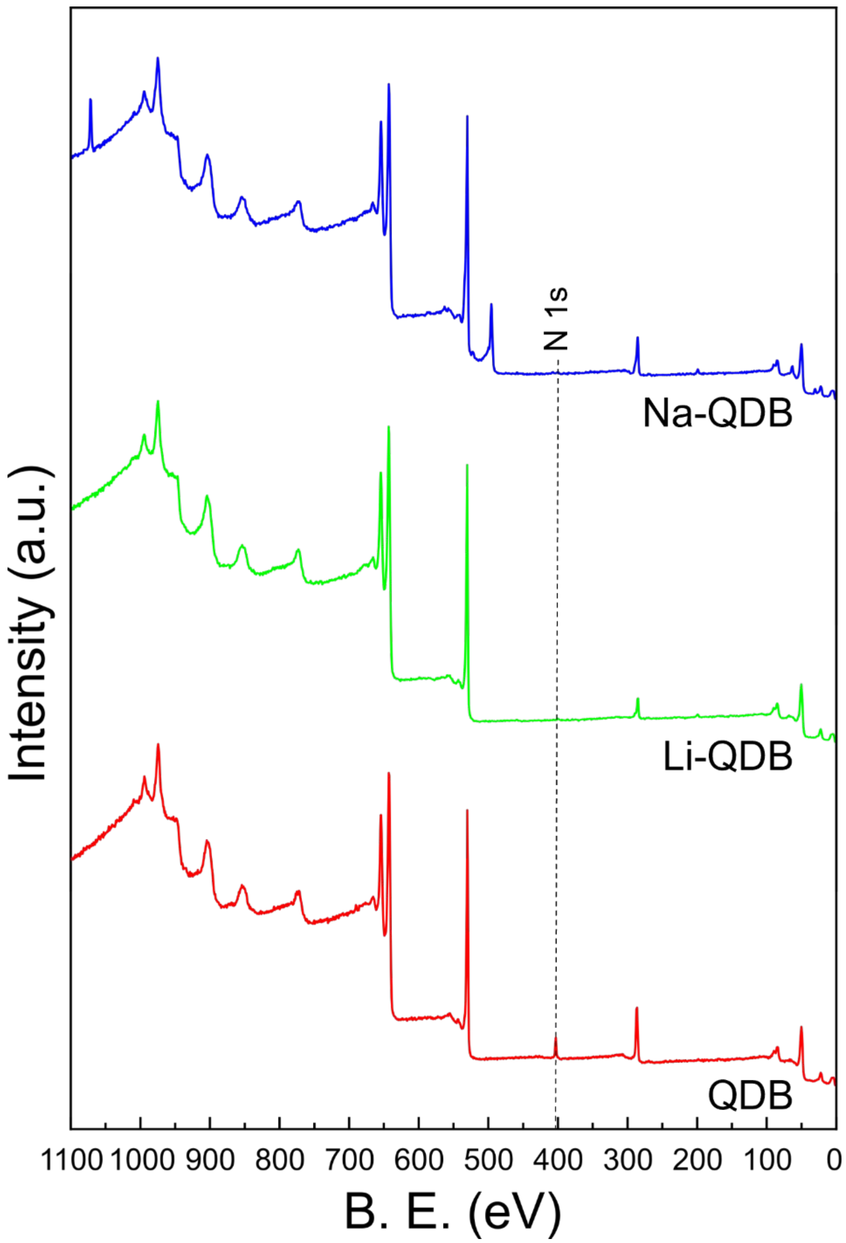

Figure S4: XPS survey spectra of EtOH-washed-QDB (red), Li-QDB (green), and Na-QDB (blue) confirm the lack of N in the ion-exchanged samples.

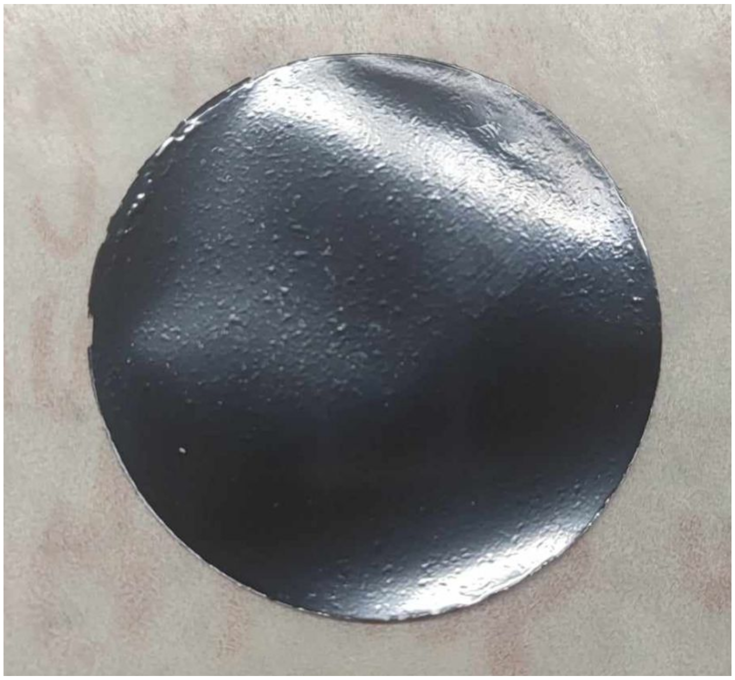

Figure S5: Filtered film of QDB colloid, without ion exchange
